# Antidepressant Drugs Effects on Blood Pressure

**DOI:** 10.3389/fcvm.2021.704281

**Published:** 2021-08-03

**Authors:** Anna Calvi, Ilaria Fischetti, Ignazio Verzicco, Martino Belvederi Murri, Stamatula Zanetidou, Riccardo Volpi, Pietro Coghi, Stefano Tedeschi, Mario Amore, Aderville Cabassi

**Affiliations:** ^1^Cardiorenal and Hypertension Research Unit, Physiopathology Unit, Clinica Medica Generale e Terapia Medica, Department of Medicine and Surgery (DIMEC), University of Parma, Parma, Italy; ^2^Department of Neuroscience and Rehabilitation, Institute of Psychiatry, University of Ferrara, Ferrara, Italy; ^3^Research Group on Mental and Physical Health of the Elderly (ARISMA), Bologna, Italy; ^4^Section of Psychiatry, Department of Neuroscience, Ophthalmology, Genetics, and Infant-Maternal Science, Istituti di Ricovero e Cura a Carattere Scientifico (IRCCS) Ospedale Policlinico San Martino, University of Genoa, Genoa, Italy

**Keywords:** arterial hypertension, orthostatic hypotension, selective serotonin reuptake inhibitors, serotonin-norepinephrine reuptake inhibitors, dopamine–norepinephrine reuptake inhibitor, tricyclic and tetracyclic antidepressants, monoamine oxidase inhibitors

## Abstract

Individuals suffering from depressive disorders display a greater incidence of hypertension compared with the general population, despite reports of the association between depression and hypotension. This phenomenon may depend, at least in part, on the use of antidepressant drugs, which may influence blood pressure through different effects on adrenergic and serotoninergic pathways, as well as on histaminergic, dopaminergic, and cholinergic systems. This review summarizes extant literature on the effect of antidepressant drugs on blood pressure. Selective serotonin reuptake inhibitors are characterized by limited effects on autonomic system activity and a lower impact on blood pressure. Thus, they represent the safest class—particularly among elderly and cardiovascular patients. Serotonin–norepinephrine reuptake inhibitors, particularly venlafaxine, carry a greater risk of hypertension, possibly related to greater effects on the sympathetic nervous system. The norepinephrine reuptake inhibitor reboxetine is considered a safe option because of its neutral effects on blood pressure in long-term studies, even if both hypotensive and hypertensive effects are reported. The dopamine–norepinephrine reuptake inhibitor bupropion can lead to blood pressure increases, usually at high doses, but may also cause orthostatic hypotension, especially in patients with cardiovascular diseases. The norepinephrine–serotonin modulators, mirtazapine and mianserin, have minimal effects on blood pressure but may rarely lead to orthostatic hypotension and falls. These adverse effects are also observed with the serotonin-reuptake modulators, nefazodone and trazodone, but seldomly with vortioxetine and vilazodone. Agomelatine, the only melatonergic antidepressant drug, may also have limited effects on blood pressure. Tricyclic antidepressants have been associated with increases in blood pressure, as well as orthostatic hypotension, particularly imipramine. Oral monoamine–oxidase inhibitors, less frequently skin patch formulations, have been associated with orthostatic hypotension or, conversely, with hypertensive crisis due to ingestion of tyramine-containing food (i.e., cheese reaction). Lastly, a hypertensive crisis may complicate antidepressant treatment as a part of the serotonin syndrome, also including neuromuscular, cognitive, and autonomic dysfunctions. Clinicians treating depressive patients should carefully consider their blood pressure status and cardiovascular comorbidities because of the effects of antidepressant drugs on blood pressure profiles and potential interactions with antihypertensive treatments.

## Introduction

The World Mental Health Survey estimates that depression affects 322 million people worldwide with an overall prevalence of 4.4% while being higher in older age groups with more than 7.5% in females and 5.5% in males aged 55–75 years (1). Depressive disorders range from 22.5% in Afghanistan to 2.5% in Japan, with the United States, Italy, France, and Germany lower than 5%, Switzerland 6.2%, and China and the United Kingdom around 3% (2).

Depressed patients display a higher incidence of hypertension compared with the general population (3, 4). The risk seems almost double among Caucasians and African Americans (5). Females have a major prevalence of the depressive disorder, but depressed men are at higher risk of developing hypertension, possibly because of an unhealthier lifestyle (1, 6, 7). Fertile women carry a lower risk vs. postmenopausal women, suggesting the involvement of sex hormones, further confirmed by the similar risk between postmenopausal women and men (8).

The increase in blood pressure (BP) among depressed individuals may involve several mechanisms, including endothelial dysfunction, low-grade inflammation, hypercoagulation (9–11), altered hypothalamic–pituitary–adrenal axis (3), and systemic autonomic dysfunction with sympathetic activation prevailing over parasympathetic drive (4, 12). Depressed patients may be less inclined to follow proper lifestyle recommendations and less adherent to antihypertensive and other drug therapies. However, major depression is not only associated with hypertension but also modest, while not significant, BP increases (13–15) or decreases have been previously reported in depressed patients (16, 17). Another possible reason for BP level abnormalities in depressed individuals is the use of antidepressant drugs, which can affect BP levels by inducing hypertension, hypotension, and orthostatic hypotension. This represents a critical issue for patients and clinicians, whether psychiatrists, internists, cardiologists, or family doctors. Several classes of antidepressant drugs may affect BP through various mechanisms, e.g., altering neural pathways involved in the vascular tone control, cardiac heart rate (HR) automatisms, or conduction mechanisms.

Given these premises, this review aims to discuss the effects on BP associated with antidepressant drugs, including selective serotonin reuptake inhibitors (SSRIs), serotonin–norepinephrine reuptake inhibitors (SNRIs), selective norepinephrine reuptake inhibitors, dopamine (DA)–norepinephrine reuptake inhibitors, norepinephrine–serotonin modulators, serotonin agonist/antagonist-reuptake inhibitors, tricyclic and tetracyclic antidepressants (TCAs), and monoamine oxidase inhibitors (MAOIs), underscoring their pharmacological properties and how this information can be used in clinical practice.

## Methods

**Figure 1:**
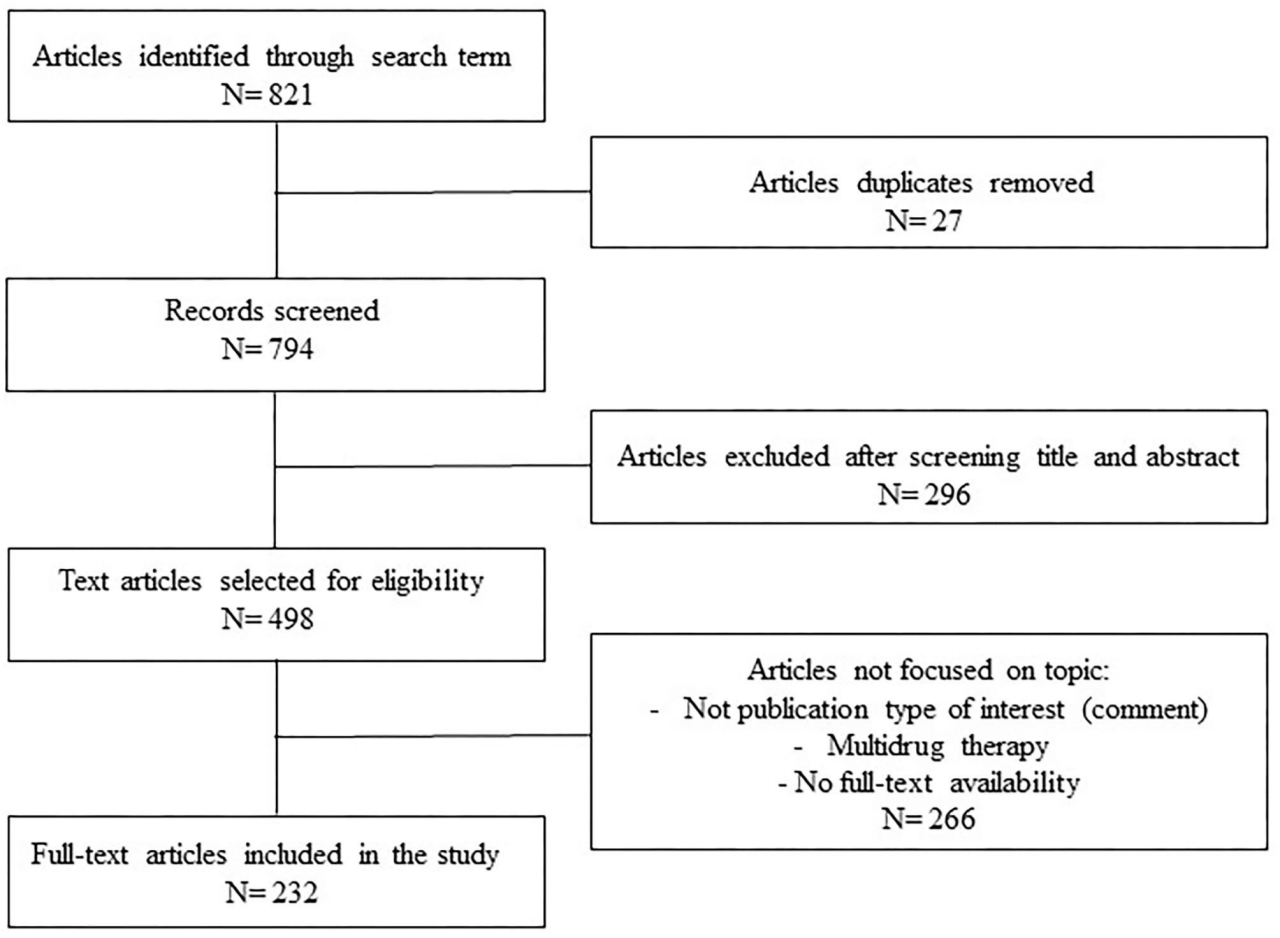
Flow chart of the selection of articles.

An extensive review of English language literature was performed to identify all relevant articles describing the BP changes induced by antidepressant drugs. We searched PubMed, EMBASETM, CINHAL, Web of Science, and Cochrane databases for relevant articles. Related search terms were used as follows: (“blood pressure, arterial hypertension” [Mesh]) OR (“antidepressant drugs” [Mesh]) OR (“orthostatic hypotension” [Mesh]) OR (“arterial hypotension” [Mesh]) OR (“major depression” [Mesh]) OR (“depressive disorders” [Mesh]) OR (“serotoninergic syndrome” [Mesh]) OR (“cheese reaction” [Mesh]) AND (“antihypertensive agents” [Mesh]) (Figure 1). Medical subject heading terms were used to enhance electronic searches. Hand searches of references identified additional studies of interest, and at least two reviewers independently reviewed each article for eligibility. We excluded conference proceedings. The search was last updated on April 26, 2021. The nomenclature of the drugs and their molecular targets conform to the recently published International Union of Basic and Clinical Pharmacology/British Pharmacological Society Guide to Pharmacology nomenclature classification (18).

### Cardiovascular Alterations in Depressive Disorders

#### Neurotransmitters Involved in Depression

5-Hydroxytryptamine (5-HT) plays a major role in the pathogenesis of depressive disorders through its action on the central nervous system, whereas it also influences the peripheral cardiovascular system. 5-HT can act on multiple pathways, including the central and peripheral autonomic nervous systems, kidney sodium and water balance, and adrenal gland hormone secretion, all of which are involved in short- and long-term BP control. 5-HT effects on BP are various and start *via* a direct arterial constriction but also include different responses depending on the type of vascular bed and on the activation of 5-HT receptor subtypes. Vasoconstriction mainly relates to 5-HT_2A_ and 5-HT_1B/1D_ receptor activation (19). The effects of 5-HT on the heart are diverse; on one side, a positive inotropic, lusitropic, and pro-arrhythmic effect is reported in humans; on the other, it can induce bradycardia by activating the parasympathetic drive and by inhibiting the release of norepinephrine (NE) (20). 5-HT also has direct renal anti-diuretic and anti-natriuretic effects, leading to increased extracellular volume (19). 5-HT also affects BP control by increasing salt and water reabsorption by stimulating cortisol and aldosterone secretion and facilitating epinephrine release from the adrenal medulla (21). Both 5-HT excitatory and inhibitory effects on the central and peripheral autonomic nervous systems have been described. 5-HT-related BP rise may depend on the central stimulation of the 5-HT_2A_ receptor, the rise of sympathetic activity, and vasopressin release (22), but it is buffered by 5-HT_1A_-mediated sympathetic inhibition and parasympathetic vagal activation leading to a fall in BP (23). NE is another neurotransmitter whose increased availability represents the target of the antidepressant treatment. In fact, in depression, NE deficit relates to decreased cognitive difficulties (i.e., decreased attention and concentration), low motivation, and low energy (24), whereas 5-HT deficit has been mainly linked to symptoms of anxiety, obsessions, suicide, and appetite disturbances (25). NE can increase BP through arterial postsynaptic α1-adrenergic receptor stimulation and causes positive myocardial chronotropic, inotropic, and lusitropic effects through a β1-adrenergic receptor activation. The hypertensive effects of NE on postsynaptic α1-adrenergic receptors are only partially counterbalanced by presynaptic α2-adrenergic receptor stimulation, resulting in a reduced release of catecholamines. DA, the precursor of NE and epinephrine, is also involved in depression, particularly in specific neural circuit activities influencing reward prediction, motivational arousal, and responsiveness to conditioned incentive stimuli (26). In fact, several symptoms observed in depression, such as anhedonia and amotivation, are associated with DA deficit, particularly in the medial frontal cortical regions and in the amygdala (27). DA affects the cardiovascular system in a dose-dependent way: at a low dose, it acts on D1- and D2-like dopaminergic receptors located in the coronary mesenteric and renal arteries and tubules, resulting in local vasodilation and increased natriuresis without inducing a drop in systemic BP (28). At high doses, DA stimulates β-adrenergic receptors and induces positive cardiac inotropic and chronotropic effects. At very high doses, α1-adrenergic receptors are also activated by DA, determining systemic vasoconstriction and BP elevation.

#### Vascular Abnormalities in Depression

In the last decade, depression has been considered a risk factor for cardiovascular disease, independent of traditional risk factors (29, 30). Major depression in otherwise healthy patients is involved in the development of adverse cardiovascular events (31). The mechanisms that link depression and cardiovascular alterations are far from clear (32). Major depression has been associated in depressed adolescents with reduced parasympathetic and increased sympathetic tone (33). In depressed patients, there is substantial evidence showing alterations in the autonomic nervous system leading to a significant reduction in HR variability. HR variability is a widely recognized prognostic risk factor for adverse cardiovascular events, such as myocardial infarction and arrhythmias, and cardiac mortality (12, 32). In addition, because depressive disorders are characterized by chronic stress, levels of circulating cortisol are increased in the long term. In this setting, immune cells become insensitive to the regulatory effects of cortisol and contribute to the chronic inflammation seen in depressed patients (34). Cortisol also increases arterial contractile sensitivity to NE and suppresses the production of prostacyclin and nitric oxide in endothelial cells, thereby resulting in higher vascular resistance (35). In patients with major depression, an increase in blood viscosity can contribute to the elevation of systemic vascular resistance (36, 37). In addition to abnormalities in resistance arteries with a rise in systemic vascular resistance, alterations are also observed in large arteries (brachial) of depressed patients (38). Carotid intima-media thickness is increased in depressed patients as well as the carotid-femoral pulse way velocity (39), both known predictors of major cardiovascular events. It seems that antidepressant drugs can affect the progression of arterial stiffness; independently from an effect on BP, it has been observed that duloxetine, an SNRI, can accelerate vascular damage while the SSRI escitalopram is neutral (40). Furthermore, a recent interesting report showed that 6-month treatments with the SSRI citalopram improved endothelial function and arterial stiffness (41).

#### Antihypertensive Therapy and Depression

Several pathogenetic mechanisms involved in BP regulation and the targets of effective and commonly prescribed antihypertensive drugs can be involved in the rise or reduction in depressive symptoms (42). Historically, patients treated with beta-adrenergic blockers have been considered at high risk for developing depression (43), and this is particularly relevant for lipophilic vs hydrophilic drugs and for non-selective drugs, as recently demonstrated (43, 44). However, a recent study based on Danish population registers showed amelioration of depressive symptoms in patients treated with propranolol, atenolol, bisoprolol, and carvedilol. Among the other antihypertensive drug classes, angiotensin-converting enzyme inhibitors (ramipril and enalapril) and calcium channel antagonists (amlodipine, verapamil alone, and verapamil combinations) also improved depressive symptoms (42) even if randomized clinical trials on renin–angiotensin system targeting agents and depression are lacking. Furthermore, it is also important to consider how the frequent concomitance of depression and arterial hypertension in patients should make us pay attention to the possible interactions between antihypertensive and antidepressant drugs. Table 1 reports the interactions between these two classes of drugs that involve both pharmacokinetic and pharmacodynamic mechanisms and that can cause serious adverse effects by enhancing or buffering the BP-lowering effect of antihypertensives, resulting in severe hypotension or bradycardia or in a BP rise.

**Table 1:** List of pharmacological characteristics of antidepressant drug class including therapeutic dose, half-life, metabolism, and antihypertensive drug interactions.

**Antidepressant**	**Therapeutic dose (starting, Mg)**	**Half-life (hours)**	**Cyp-450 metabolizer (S)**	**Interactions with antihypertensive drugs**
**SSRI**				
Citalopram	20–40 (20)	23–45	3A4, 2C19	
Escitalopram	10–20 (20)	27–32	–	
Paroxetine	20–50 (20)	24	2D6	↑ Metoprolol (45–47), ↑ Propranolol (48)
Fluoxetine	20–80 (20)	48–72	2D6, 3A4	↑β-Blockers (48–50)
Fluvoxamine	50–300 (50)	9–28	3A4, 1A2, 2C9/2C19	↑ Carvedilol (51), ↓ Propranolol (50)
Sertraline	50–200 (50)	22–36	2D6, 3A4	↑ Carvedilol (51)
**SNRI**				
Venlafaxine	75–225, max 375 (37.5)	11	2D6, 3A4	↓ Metoprolol (52)
Desvenlafaxine	50–100 (50)	9–13	3A4	
Duloxetine	40–60, max 120 (40)	12	2D6, 1A2	↑ Metoprolol (52), ↑ Propranolol (48)
Milnacipran	30–200 (12.5)	8	3A4	
Levomilnacipran	40–120 (20)	12	3A4	
**REBOXETINE**	4–8, max 10 (4)	12	3A4	
**DNRI**				
Bupropion immediate, sustained,	225–450 (75), 200–450 (100)	8–10, 12, 24, 21	2B6	↑ Carvedilol (51), ↑ Metoprolol (52), ↑ Propranolol (48)
Extended release, hydrobromide	150–450 (150), 174–522 (174)			
**NSM**				
Mirtazapine	15–45 (15)	20–40	2D6, 1A2	↓ Clonidine (53)
Mianserin	30–60, max 90 (30)	12–29	2D6	↑ Prazosin (54)
**SARI**				
Nefazodone	100–300 (100)	2–4	3A4	
Trazodone	150–600 (150)	3–6	3A4	↑ Clonidine (55, 56)
Vortioxetine	5–20 (5)	66	2D6,3A4,2C19,2C9,2A6,2C8,	
Vilazodone	20–40 (10)	25	3A4, 2C19, 2D6	
**AGOMELATINE**	25–50 (25)	1–2	1A2	
**TCA**				
Imipramine	50–150, max 300 (25)	19	2D6, 1A2	↑β-Blockers (49, 57)
Amitriptyline	50–150, max 300 (25)	10–28	2D6, 1A2	
Clomipramine	10–200, max 250 (25)	17–28	2D6, 1A2	
Desipramine	100–200, max 300 (25)	24	2D6, 1A2	As effect of class: ↓α2-Agonists (50, 55, 57), ↑ Propranolol (57),
Nortriptyline	70–150, max 300 (25)	36	2D6	↓ Calcium Antagonists (57)
Doxepin	75–150, max 300 (25)	24	2D6	
Trimipramine	50–150, max 300 (25)	7–24	2D6, 2D19, 2C9	
Protriptyline	15–40, max 60 (15)	74	2D6	
Maprotiline	70–150, max 225 (25)	51	2D6	
**MAOI**				
Tranylcypromine	30–60 (10)	240	2A6	
Phenelzine	45–75, max 90 (15)	240	–	The first three drugs: ↓α2-Agonists (55, 57), ↑ Propranolol (57)
Selegiline patch	6–12 (6)	24	2B6, 2D6, 3A4	
Isocarboxazid	40–60 (20)	240	–	
Moclobemide	300–600 (300)	24	2C19, 2D6	

*↑ = increasing action, ↓ = reduced action of the drug*.

### Selective Serotonin Reuptake Inhibitors

SSRIs are most frequently used as first-line treatment of major depression (Table 1) (58). Their efficacy has also been demonstrated in the treatment of generalized anxiety, panic, social anxiety, and obsessive–compulsive disorders. This class includes citalopram, escitalopram, paroxetine, fluoxetine, fluvoxamine, and sertraline. Sertraline and paroxetine have also been approved for the treatment of post-traumatic stress disorder.

SSRIs selectively block presynaptic reuptake mediated by serotonin transporters (SERTs), thereby enhancing and prolonging serotonergic neurotransmission. Other effects of SSRIs are related to presynaptic serotonin (5-HT) receptor desensitization, especially 5-HT_1A_, thus facilitating 5-HT release (55). Free circulating 5-HT levels are highly variable, being mainly taken up by SERT in platelets (19). Sertraline, fluvoxamine, and paroxetine induce a 5-HT depletion of platelets after chronic treatment and an increase of 5-HT free circulating levels, potentially interfering with BP control (59). Another relevant effect of chronic SSRI treatment on the autonomic balance is represented by the increase in HR variability resulting in better clinical outcomes with reduced morbidity and mortality in patients with previous cardiac disease (Table 2) (12). Caution should be considered, especially among elderly patients in relation to SSRI-mediated vasopressin release, which might lead to the development of hyponatremia (60). Because of 5-HT-mediated pulmonary arterial vasoconstriction and smooth muscle cell proliferation, SSRIs used in the second half of pregnancy have been related to a higher risk of persistent pulmonary hypertension in newborns (61). In animal models, decreased BP levels are reported after long-term *in vivo* 5-HT administration, indicating 5-HT-mediated nitric oxide release as a mechanism involved in the fall in BP (18, 62). This puzzling picture of potential 5-HT-related short- and long-term BP effects should be considered in patients treated with SSRIs.

**Table 2:** Influence of antidepressant drugs on blood pressure and heart rate.

**Antidepressant**	**Hypertension**	**Orthostatic hypotension**	**Tachycardia**	**Bradycardia**
**SSRI**				
Citalopram	0	0	0	+ (53)
Escitalopram	0	0	0	+ (53)
Paroxetine	0	0	0	+ (53)
Fluoxetine	0	0	0	+ (53)
Fluvoxamine	0	0	0	+ (53)
Sertraline	0	0	0	+ (53)
**SNRI**				
Venlafaxine	++/+++^a^	+ (63–66)	++	0
Desvenlafaxine	++	+ (53)	++	0
Duloxetine	+	+	+	0
Milnacipran	++	0	+	+^b^ (67)
Levomilnacipran	++	0	+	0
**REBOXETINE**	0	0	+	0
**DNRI**				
Bupropion immediate, sustained, extended release, hydrobromide	++	+	+ (68–70)	0
**NSM**				
Mirtazapine	0	+	0	0
Mianserin	0	++	0	0
**SARI**				
Nefazodone	0	+	0	+ (71)
Trazodone	0	+++	0	+ (53)
Vortioxetine	0	0	0	0
Vilazodone	0	0	0	0
**AGOMELATINE**	+^b^	0	0	0
**TCA**				
Imipramine	+	++++	++	0 (72)
Amitriptyline	+	+++	+++	0
Clomipramine	+	++	++	0
Desipramine	+	++	+	0
Nortriptyline	+	+	+	0
Doxepin	+	++	++	0
Trimipramine	+	+++	++	0
Protriptyline	+	++	+	0
Maprotiline	+	++	0	0
**MAOI**				
Tranylcypromine	+++^c^	++	+++^c^	0 (72)
Phenelzine	+++^c^	++	+++^c^	0
Selegiline patch	0	0	0	0
Isocarboxazid	+++^c^	+	+++^c^	0
Moclobemide	+++^c^	0	+++^c^	0

*Effects: ++++ = high, +++ = moderate, ++ = low, + = very low, 0 = none;*

a *Dose higher than 225 mg/day;*

b *Overdose;*

c *When co-administered with tyramine-containing food*.

#### Citalopram, Escitalopram, and Paroxetine

Chronic treatment with citalopram and escitalopram, its therapeutically active S-enantiomer, did not change systolic and diastolic BP levels (BP values varying between 0.3 and <1 mmHg) nor HR neither QT interval length in two studies on elderly depressed patients, with and without coronary artery disease (73, 74). In a geriatric population of patients, citalopram was effective in further reducing BP when depressed and hypertensive subjects were treated with amlodipine (75). Non-significant effects of citalopram on BP levels (<1 mmHg) were also reported in a similar group of geriatric patients (76). Escitalopram in hypertensive and depressed individuals younger than 65 years did not show significant effects on BP (difference <1 mmHg), but a decrease in HR (7 bpm) was reported (77). Escitalopram, citalopram, and the other SSRIs (fluoxetine, sertraline, and paroxetine) did not significantly change BP levels in young depressed female patients without any other metabolic or hypertensive comorbidities (<3 mmHg) (78). Paroxetine, different from other SSRIs, is characterized by a mild anticholinergic and inhibitory effect on the NE transporter (NET). In depressed patients with ischemic heart disease, however, paroxetine did not show significant effects on HR, conduction intervals or ventricular arrhythmias, diastolic supine and standing BP, and standing systolic BP. However, a significant increase in supine systolic BP (4 mmHg) was observed after 6 weeks of treatments (79). In mentally healthy subjects with a history of coronary disease undergoing periods of psychological stress, paroxetine lowered systolic and diastolic BP by 10–15% from baseline levels in non-stressful times (80). Similar results were observed in active smokers (5-mmHg reduction of systolic BP) (81). Its adverse effects are similar to other SSRIs, but as a weak antimuscarinic drug, constipation, dry mouth, and sedation are reported. Patients with renal and hepatic impairment should be administered a lower dose when titrating the drug. Paroxetine has a relevant inhibitory capacity on CYP2D6 metabolic activity and, when combined with metoprolol, can significantly increase metoprolol circulating levels (3- to 5-fold) and reduce the HR and systolic BP of patients, both in rest and exercise state. Therefore, a dose adjustment, close monitoring of the metoprolol-related adverse effects, or a change in the antidepressant drug is needed (Table 1).

#### Sertraline, Fluvoxamine, and Fluoxetine

Sertraline, a widely used SSRI, has a moderate ability to block the DA reuptake transporter. This leads to increased DA neurotransmission, which might contribute to its therapeutic action. Normally, increasing the levels of 5-HT is accompanied by reductions in DA release. In addition to effects on 5-HT and DA pathways, some SSRIs, including sertraline, fluvoxamine, escitalopram, and citalopram but not paroxetine, possess a high-to-moderate affinity for the sigma-1 receptor, suggesting that some of their antidepressant effects may depend on this pathway. Sigma-1 receptors have been found in limbic and other cerebral endocrine areas such as the hippocampus, frontal cortex, and hypothalamus, which have been implicated in the pathophysiology of depression (82). Sigma receptors were initially considered a subclass of the sigma opioid receptors and included two subtypes: sigma-1 and sigma-2. Animal and human studies show that agonists of the sigma-1 receptor cause vasodilation and systolic BP fall due to nitric oxide release from endothelial and neuronal nitric oxide synthases. This suggests a potential application of sigma agonists in antihypertensive drug treatments (83, 84). Such effects may be especially relevant for fluvoxamine, fluoxetine, and escitalopram, which are strong agonists of the sigma-1 receptor but not sertraline, which, despite a high affinity for the sigma-1 receptor, only acts as a mild antagonist, making its effect on BP overall low (83). In fact, sertraline rarely causes arterial hypotension and is considered safe even in patients with recent myocardial infarction or unstable angina. Sertraline was indistinguishable from placebo across all surrogate measures of cardiovascular safety, including change in BP (not significant increases of 3 and 2 mmHg for systolic and diastolic BP levels, respectively), HR, arrhythmias, and left ventricular ejection fraction (85). Sertraline was found to be safe, well-tolerated, and effective even in elderly patients suffering from hypertension and other forms of vascular comorbidity: a significant increase in both standing systolic (+6.5 mmHg) and diastolic BP (+3.8 mmHg) but not in supine BP (86). Fluvoxamine, possibly acting on sigma-1 receptors, can induce sedation and fatigue in some patients but usually does not cause postural hypotension nor other significant cardiac effects except for slight bradycardia. Similarly, it carries a very low risk of QT interval prolongation (87). Fluoxetine, in addition to SSRI effects, has antagonist effects on 5-HT_2C_ receptors, which could lead to increases in both NE and DA neurotransmissions. A modest reduction in sitting and standing systolic (−2.9 and −2.6 mmHg) and diastolic BP (−2.3 and −1.5 mmHg) was observed after short periods (12 weeks) of treatment with fluoxetine in major depressive patients normotensive and hypertensive. A diversified pattern between hypertensive and normotensive subgroups was reported with hypertensive (diastolic BP between 90 and 95 mmHg) patients showing a larger BP reduction (−4.3 and −5.6 mmHg sitting and standing systolic BP and −4.8 and −3.9 mmHg for diastolic BP) than in normotensive with pretreatment diastolic BP values between 60 and 90 mmHg (−4.3 and −5.6 mmHg sitting and standing systolic BP and −4.8 and −3.9 mmHg for diastolic BP). The normotensive subjects with pretreatment diastolic BP < 60 mmHg had a modest increase in BP levels instead (88). Moreover, fluoxetine does not cause changes in BP in patients with depression and recent myocardial infarction (89). Sertraline, fluvoxamine, and fluoxetine do not require dose adjustments in patients with impaired renal function, but it may be necessary to reduce doses in those with hepatic impairments. In a large meta-analysis evaluating the effect of SSRIs on BP in depressed patients, no significant differences in systolic and diastolic BP were observed vs. placebo. No statistical difference was also reported for five widely used SSRIs (paroxetine, fluoxetine, sertraline, escitalopram, and citalopram) (90). In general, SSRIs did not significantly affect BP levels and are considered safe in patients with and without preexisting cardiovascular disease (Table 2) (73). A recent review confirmed the safety of the SSRI drug class but reported the risk of developing orthostatic hypotension at high dosage (91). This claim should, however, be tempered with the risk of serotoninergic syndrome, a rare potential life-threatening condition occurring when SSRIs are used at high doses or combined with other 5-HT agonists. The massive release of 5-HT leads to activation of central 5-HT_1A_ and 5-HT_2A_ receptors (92) and induces neuromuscular hyperactivity (myoclonus, hyperreflexia, and rhabdomyolysis), cognitive dysfunction (agitation and confusion), autonomic symptoms (hyperthermia, tachycardia, and hypertensive crisis), and, in rare cases, multi-organ failure syndrome (19, 92, 93). Posterior reversible encephalopathy syndrome has also been reported as a complication of severe hypertension (94). Treatments include withdrawal of the drug(s) and supportive care, benzodiazepines for the control of agitation and convulsions, cyproheptadine (a histamine-H1 and 5-HT_2A_ antagonist and widely used antidote for neuromuscular paralysis), and sedation and intubation in case of severe muscle rigidity and hyperthermia (92, 95).

### Serotonin–Norepinephrine Reuptake Inhibitors

SNRIs include venlafaxine, desvenlafaxine, duloxetine, milnacipran, and levomilnacipran (Table 1). The first three compounds mainly act on serotoninergic neurons inhibiting presynaptic 5-HT, whereas milnacipran and levomilnacipran preferentially block the reuptake of NE. SNRIs can indirectly affect the dopaminergic system by increasing synaptic DA levels in the prefrontal cortex because presynaptic NET inhibition also acts on DA reuptake (55). This class of drugs may increase BP (11, 15, 19), in particular diastolic BP (16, 90), through an increased myocardial and vascular sensitivity to sympathetic stimulation, which results in a rise in cardiac output and ultimately an increase in BP (Table 2) (96). Antidepressants, particularly SNRIs, require special caution when they are prescribed to pregnant women before the 16th week of gestation because of the risk of gestational hypertension and preeclampsia (97–99).

#### Venlafaxine, Desvenlafaxine, and Duloxetine

Venlafaxine is indicated as a treatment for major depressive disease, generalized anxiety disorder, panic disorder, and social anxiety disease. Its half-life is 5 h, but its major and effective metabolite (O-desmethylvenlafaxine) has an 11-h half-life (Table 1). This metabolite has a greater effect on NET than SERT compared with the parent compound (55). The typical starting dose of venlafaxine is 37.5 mg, which, when tolerated, can be slowly increased up to 375 mg once daily. At lower doses, the inhibition of 5-HT reuptake prevails, whereas at high doses (over 225 mg) venlafaxine mainly acts as a NE reuptake inhibitor (100, 101). Desvenlafaxine can block both 5-HT and NE reuptake at low doses (102, 103). The immediate-release formulation of venlafaxine can induce sustained diastolic hypertension in 10–15% of patients with a lower risk of raising BP with the extended-release formulation. The extended-release formulation of venlafaxine has been related to BP elevation in almost 6% of patients, whereas orthostatic hypotension is even three times more frequent; it ought to be noted that two of three of those who developed orthostatic hypotension took cardiovascular drugs, and 44% were treated with benzodiazepines (104). Chronic venlafaxine treatment not only determines a sustained increase in BP (diastolic BP increase up to 15 mmHg) (105) but can also facilitate the occurrence of hypertensive crisis in normotensive patients as well (53, 106–108). It has been shown that venlafaxine does not worsen preexisting hypertension but causes a rise in BP in normotensive patients (109). Desvenlafaxine, even at low doses, can increase HR and BP (0.66–3.41-mmHg rise in supine diastolic BP and 2-mmHg rise in supine systolic BP at lower doses) (102, 110). Moreover, the incidence of hypertensive crisis is described in adults (53, 103) but not in children and adolescents (111). Posterior reversible encephalopathy syndrome has also been described as a result of severe hypertension after starting moderate doses of venlafaxine (112). Because of a potential increase in BP, venlafaxine has also been used to treat symptomatic hypotension (113), even if its use is not approved for the treatment of orthostatic hypotension in the last 2017 American College of Cardiology/American Heart Association/Heart Rhythm Society Syncope Guidelines and 2018 European Society of Cardiology Guidelines for the diagnosis and management of syncope (114, 115).

Duloxetine is prescribed for major depressive disorder as well as diabetic peripheral neuropathic pain, chronic musculoskeletal pain, and fibromyalgia (116). Duloxetine can increase HR and systolic and diastolic BP (+1.89 mmHg of diastolic BP) (117–119); a marked increase in BP levels was reported when supratherapeutic doses were used (+12 mmHg for systolic BP and +7 mmHg for diastolic BP) (90). Less than 1% of patients develop hypertensive crises with duloxetine (53). However, it was also reported that the effect of duloxetine on BP is clinically negligible (<1-mmHg rise for both diastolic and systolic BP) in another study, indicating this drug as a safe option for patients with cardiovascular disease (120). Sporadic Takotsubo cardiomyopathy has also been reported after duloxetine and venlafaxine use (121, 122). Venlafaxine and duloxetine treatment has also been correlated to orthostatic hypotension in young and old patients at therapeutic doses (63, 123–125). Increased synaptic NE levels by venlafaxine or duloxetine can stimulate presynaptic α-2 adrenergic receptors, thus reducing NE release from the nerve terminals. Alpha-2 adrenergic receptors are polymorphic, and some specific genetic variants show a higher propensity for reduced NE release from the nerve endings after activation of α-2 adrenergic receptors (126–128). In addition, higher plasma venlafaxine levels are found in the poor metabolizer genotype CYP2D6, leading to sustained stimulation of α-2 adrenergic receptors by NE (125). Both these mechanisms may participate in causing orthostatic hypotension (Table 2).

A recent review shows a low risk for venlafaxine to induce a rise in BP, and, on the contrary, a decreased or neutral effect of this drug on BP was reported more frequently (124). Another study confirmed no effect on the cardiovascular system by venlafaxine in geriatric patients (76).

#### Milnacipran and Levomilnacipran

Milnacipran is prescribed not only for major depressive disease but also for fibromyalgia, and it can be an effective treatment for chronic fatigue syndrome and anxiety (Table 1) (129). Levomilnacipran is the enantiomer of the racemic drug, milnacipran, and its affinity for NET is higher than other SNRIs (with a NET/5-HT transporter ratio of 1.5 in humans) (130). Its serotoninergic activity increases when administered at higher doses (131). Milnacipran and levomilnacipran are contraindicated in end-stage kidney disease and chronic liver disease and patients with previous cardiovascular disease. Milnacipran and levomilnacipran can induce an increase in both systolic and diastolic BP (+3 and +3.2 mmHg in systolic and diastolic BP, respectively, for levomilnacipran, and + 3 mmHg for both in systolic and diastolic BP with milnacipran) (132–137) as observed during office and ambulatory BP monitoring (138–140). Takotsubo cardiomyopathy has been described at both therapeutic doses and in overdoses of milnacipran; high doses of this drug show negative inotropic, chronotropic, and dromotropic effects (67, 141).

### Selective Norepinephrine Reuptake Inhibition

Reboxetine is prescribed for major depressive disease, dysthymia, and attention-deficit/hyperactivity disorder, with better tolerability than TCA (Table 1) (142). Its antidepressant effects relate to the sustained increase in NE levels in the central nervous system (143, 144). The effects of reboxetine on the cardiovascular system are diverse when comparing acute with chronic treatments. Reboxetine has a highly selective action on NET, a lower affinity on muscarinic, histamine-H1, and adrenergic α1 receptors (145) and can increase DA levels through presynaptic NET inhibition (143). Neutral effects on BP have been reported in long-term treatment with reboxetine (Table 2) (142, 146), whereas a transient BP reduction has been reported after acute administration (-2.1 mmHg) (147). In fact, acute administration of reboxetine can lower BP by presynaptic α2-adrenoceptor stimulation resulting in NE release inhibition (147). Desensitization of α2-adrenoceptors occurs after long-term treatment, thus facilitating NE release (143, 147) and leading to reduced sympathetic outflow and neutral effects on BP in both human and animal studies (144, 147). In short-term studies, reboxetine has been associated with an increase in HR (148, 149). A few studies in healthy subjects reported an increase in systolic (8 mmHg) and diastolic BP (4 mmHg) after reboxetine administration (148–150); conversely, arterial hypotension is observed in up to 12% of depressed patients (151). In addition, it is reported that long-term reboxetine treatment showed a decrease in systolic BP levels in patients with metabolic syndrome but an increase in lean and healthy controls (152). Therefore, the cardiovascular effects of reboxetine are variable in relation to acute or prolonged administration; even in prolonged treatment where the main results indicate a neutral effect on BP, modest phenomena of both hypotension and arterial hypertension can be observed while still considering the drug a safe option (144, 147).

In fact, according to the UK Medicines and Healthcare Products Regulatory Agency and the European Medicines Agency, both hypotension and hypertension have been reported as common undesirable effects observed in post-marketing data ^1^.

Atomoxetine is a selective inhibitor of NET and is prescribed in clinical practice for attention-deficit/hyperactivity disorder and in neurogenic orthostatic hypotension of multisystemic atrophy because of its ability to increase BP (153).

### Dopamine–Norepinephrine Reuptake Inhibitor

Bupropion is a unicyclic aminoketone drug that enhances the release of NE and DA through NET and DAT inhibition. It is used to treat major depressive disease and for smoking cessation because of its activity as a non-competitive antagonist of nicotinic acetylcholine receptors. Because of renal and hepatic elimination, low doses should be used in patients with renal or hepatic failure (Table 1). Elevated plasma concentrations of bupropion can also lower epileptic thresholds. Chronic bupropion treatment causes a significant rise in diastolic BP in depressed outpatients (+7 mmHg) (154), whereas, in hospitalized smokers with an acute cardiovascular disease, no statistically significant differences were observed in both systolic (+8.7 mmHg) and diastolic BP (+7.4 mmHg) when compared with placebo (Table 2) (155). A neutral effect was reported in other studies (156, 157). When patients were treated with high doses (300–400 mg/day), a significant rise in BP was observed instead (68). In cases of bupropion overdoses, hypertension developed in 1 to 4% of patients, whereas hypotension was reported in 1–2% (158). Bupropion poisoning was linked to mild hypertension in 56% of cases (69). There is also a report of supine hypertension (+5 mmHg for systolic and +3 mmHg for diastolic BP) associated with orthostatic hypotension (with a mean decrease of 4 mmHg, up to a maximum value of −40 mmHg) after bupropion treatment in depressed coronary heart disease patients (159).

### Norepinephrine–Serotonin Modulators

Mirtazapine is approved for the treatment of major depressive and generalized anxiety disorders. It is frequently prescribed at bedtime because of its efficacy in sleep induction. It acts as an α2-adrenergic, 5-HT_2A_, 5-HT_2C_, 5-HT_3_, histamine-H1, and H2 receptor antagonist. It should be prescribed with caution in case of renal, cardiac, and hepatic impairments (Table 1). Mianserin has a tetracyclic structure and has the same pharmacological profile as mirtazapine but with a more pronounced antagonism of α1-adrenergic receptors activity (Table 1) (160). Both drugs can induce weight gain, but orthostatic hypotension is more frequent with mianserin (fall in BP up to 25 mmHg in hypertensive patients) (91, 160, 161). Although mirtazapine is reported to determine orthostatic hypotension in 7% of treated patients (162), there are also other studies in the literature showing no effect on BP (163, 164). It has been recently considered a safe drug for cardiovascular patients (124). Mianserin, by decreasing total vascular resistance, is often responsible for decreasing systolic and diastolic BP while standing (165), even if the neutral effect on BP changes is also reported (166, 167). Orthostatic hypotension is less common if the dosage of mianserin is raised gradually (168), in contrast, to rapidly increasing the dose (until 60 mg), which can induce a marked reduction in BP (Table 2) (169).

### Serotonin Agonist/Antagonist-Reuptake Inhibitors

Nefazodone, trazodone, vortioxetine, and vilazodone act as agonist/antagonist of 5-HT receptors and SERT inhibitors (Table 1). Nefazodone is prescribed for major depressive disorders, and its activity is related to 5-HT_2A_ receptor antagonism and on SERT inhibition (55). Nefazodone and its major metabolite hydroxyl-nefazodone pose several safety concerns, particularly liver toxicity. A reduction in systolic BP (up to −7 mmHg at higher doses) and HR has been reported in patients treated chronically (at least 3 weeks) with nefazodone *via* serotonin-mediated inhibition of sympathetic cardiac and vascular tone (Table 2) (170). In experimental models, 5-HT reduces HR *via* a centrally related increase in vagal tone by 5-HT_1A_ receptor stimulation, post-ganglionic cholinergic nerve endings by 5-HT_3_ receptor activation, and through the presynaptic inhibition of sympathetic drive (170). In case of overdoses, BP changes are not frequent: arterial hypotension affects up to 1.6% of cases, whereas hypertension is rarer (0.4%) (71).

Trazodone is used at low doses for insomnia, especially among the elderly, whereas its antidepressant activity appears at higher doses (150 mg/day). At low doses, trazodone acts mainly as a potent 5-HT_2A_ receptor antagonist but a low H1-histamine receptor and α1-adrenergic antagonist; at high doses, SERT inhibition and 5-HT_2C_ antagonism are the drug's targets (55, 171). Caution should be used in patients with hepatic or cardiac dysfunction because of potential arrhythmogenic effects (QT prolongation). A case of fatal overdose due to torsades de pointes was also reported (172). Orthostatic hypotension occurs when elderly or patients with preexisting heart disease are treated with trazodone and is related to α1-adrenergic antagonism (171, 173, 174). Severe hypotension, even unresponsive to volume repletion, is described in cases of drug overdoses (over 2 g) (Table 2) (175).

Vortioxetine is a recently marketed antidepressant, which is indicated for major depression, particularly when associated with cognitive dysfunction (Table 1). It acts in multimodal ways, being a 5-HT_3_, 5-HT_7_, and 5-HT_1D_ receptor antagonist, a 5-HT_1B_ partial agonist, a 5-HT_1A_ receptor full agonist, and a SERT inhibitor. Vilazodone, another antidepressant launched 7 years ago, is a 5-HT_1A_ partial agonist and a SERT inhibitor; it is indicated for major depressive disorders, anxiety disorder, and obsessive–compulsive disorder (Table 1). Concerning their cardiac and vascular profiles, vortioxetine and vilazodone are considered safe, with non-relevant effects on BP and a cardiovascular profile comparable with placebo (Table 2) (55, 176–180).

### Serotonin Antagonist–Melatonin Agonist

Agomelatine is a melatonin receptor, MT1 and MT2 agonists, and a 5-HT_2B_/5-HT_2C_ receptor antagonist prescribed for depression. It is proven to be effective at relieving the sleep disturbances that are often associated with depression (Table 1). In fact, its action on MT1 and MT2 receptors located in the suprachiasmatic nucleus of the hypothalamus can regulate the circadian rhythm and influence the autonomic output to the cardiovascular system. Its antagonism of 5-HT_2B_/5-HT_2C_ receptors contributes to suppressing the secretion of melatonin by the pineal gland during light hours (55). Its use can be complicated by hepatotoxicity at high doses. Agomelatine seems to be a safe drug from a cardiovascular point of view (124) and has several beneficial anti-inflammatory, antioxidant, and antihypertensive properties (91). Similarly, chronic evening treatment with melatonin, which is closely structurally related to agomelatine, reduces daytime BP of hypertensive patients and induces coronary vasodilation (62, 181, 182). However, a few reports indicate an increase in BP when high doses are administered or in slow cytochrome P450 2C19 metabolizers (Table 2) (183).

### Tricyclic and Tetracyclic Drugs

These drugs are used to treat depression, panic attacks, generalized anxiety disorder, post-traumatic stress disorder, bulimia nervosa, smoking cessation, and chronic pain states. Because their mechanism action targets multiple pathways (mainly acting as SERT and NET inhibitors and/or as H1-histamine, α1-adrenergic, 5-HT_2_, and M1-cholinergic receptor antagonists), a wide spectrum of adverse effects can complicate their use. Tricyclic compounds include imipramine, amitriptyline, clomipramine, doxepin, trimipramine, desipramine, nortriptyline, and protriptyline (Table 1). The tetracyclic maprotiline differs from the others because of an adjunctive ring in the central structure (Table 1). They can affect BP levels by various mechanisms. First, the increase in systolic and diastolic BP levels has been ascribed to the anticholinergic effects of these drugs (16, 53). Orthostatic hypotension, however, is reported more often than increased BP and relates to their antagonism of α1-adrenergic receptors (Table 2) (124, 184–190). The increased occurrence of falls in the elderly treated with TCA can be attributed to orthostatic hypotension combined with sedation linked to the histamine–H1 receptor antagonism effect of the drugs (184). Arterial hypotension, not only orthostatic hypotension, is explained in chronic users of TCA by the downregulation of postsynaptic β-adrenergic receptors and their reduced responsiveness to catecholamines (191). However, long-term TCA therapy is also characterized by lower presynaptic α2-adrenergic receptor sensitivity (192), interfering with the hypotensive effect of the antihypertensive α-2 adrenergic agonist clonidine (Table 1) (52). In the case of TCA overdose, arterial hypotension represents a major clinical concern (Table 2) (193, 194): decrease in myocardial contractility, excessive vasodilation of resistant arteries (195), central nervous system depression, and decreased neuronal firing rate *via* presynaptic negative feedback mechanisms on NE release (196) are all involved. Severe hypotension can be treated with fluid resuscitation and alkalization or, if necessary, by using amine vasopressors (193). Other classes of antidepressants are preferred to TCA because of a safer cardiovascular profile; TCA not only shows changes in BP but can also facilitate arrhythmias, QT interval prolongation, and heart failure (197).

#### Imipramine, Amitriptyline, Clomipramine, Desipramine, Doxepin, and Protriptyline

Imipramine is the oldest drug in this class. It elicits a more potent action than amitriptyline as a SERT than as a NET inhibitor. Orthostatic hypotension is an important complication of imipramine (−26 mmHg of systolic BP) (198–200), amitriptyline, clomipramine (with a decrease of systolic BP of 5–10 mmHg), and its metabolite, desmethylclomipramine (201, 202), especially in older patients (203) and when doses are rapidly up-titrated (Table 2) (202, 204–206). However, other reports found no changes, or only minor effects (149, 207, 208), and even an increase in supine BP mainly after long-term treatment (209). Desipramine, nortriptyline (the active metabolite of amitriptyline), protriptyline, and maprotiline inhibit NET more than SERT. No significant effects on sitting BP levels were reported with these drugs, even if attention should be paid to the occurrence of orthostatic hypotension (−10 and −7.7 mmHg for systolic BP for hypertensive and normotensive patients treated with nortriptyline) (198, 210–213) and reduced clonidine-mediated antihypertensive effects (Table 1) (211). In experimental models of hypertension, it appears that changes in desipramine sensitivity to inhibit NET between prehypertensive and hypertensive phases can contribute to the rise in BP observed in the later phase (214). Doxepin and trimipramine act as both 5-HT and NE reuptake blockers but with a major effect on serotoninergic activity. Amitriptyline, clomipramine, doxepin, and nortriptyline have an α1-adrenergic receptor antagonist effect similar to the antihypertensive drug doxazosin, thus explaining the possible orthostatic hypotension effect, whereas protriptyline, which exhibits weaker antagonism, has a lower orthostatic hypotension rate (Table 2) (215).

### Monoamine Oxidase Inhibitors

The use of iproniazid, a non-selective, irreversible MAOI, originally prescribed for tuberculosis but found to have strong antidepressant effects, opened wide the clinical use for this class of drugs, suggesting that monoamine neurotransmission deficiencies play a central role in the development of major depression (216). Tranylcypromine, phenelzine, moclobemide, isocarboxazid, and selegiline are known as powerful antidepressants and effective agents for panic disorder and social phobia, with selegiline also being used in the early phases of Parkinson disease (Table 1). The adverse effects of MAOIs are common and consist of sexual dysfunction, constipation, sedation, dry mouth, weight gain, and insomnia, but particular concerns are the risk of “cheese reaction” and serotoninergic syndrome. The risk of developing the “cheese reaction” during treatment with MAOIs depends on the concurrent consumption of meals containing tyramine or sympathomimetic drugs (Table 3). Tyramine is normally metabolized by MAO-A located on the gut wall and by MAO-B in the liver; if MAO-A is inhibited, the bioavailability of tyramine is increased, which leads to an excess in NE, resulting in a hypertensive crisis (55, 217). Currently, they are not first-line antidepressant medications, and their use is limited to treatment-resistant or atypical depression.

**Table 3:** Dietary guidelines for patients treated with MAOIs and drugs that boost norepinephrine, must to be used cautiously in patients taking MAOIs.

**Foods to avoid (not necessary if 6 mg transdermal Selegiline is used)**	**Drugs to used cautiously**
Aged cheese	Decongestants (phenylephrine, pseudoephedrine)
Broad bean pods	Stimulants (amphetamine, methamphetamine, cocaine, modafinil)
Banana peel	Antidepressants releasing NE (SNRI, reboxetine, TCA)
Dried, aged, smoked fish	Local anesthetics containing vasoconstrictors
Soy product/tofu	Tapentadol
Sauerkraut/kimchee	Phentermine
Tyramine-containing nutritional supplement	

#### Tranylcypromine and Phenelzine

Tranylcypromine and phenelzine are non-selective irreversible MAOI, with hepatic metabolism and renal excretion (218). Tranylcypromine and phenelzine can induce a dose-related orthostatic hypotension phenomenon (219), probably related to a relaxing effect on vascular smooth muscle (Table 2) (220). Both drugs can induce hypertensive crisis-related myocardial injury due to concomitant consumption of soft cheese (Table 3) (221, 222). Patients with hepatic and cardiac disease should avoid both drugs. Phenelzine induces more weight gain and sedation than other MAOIs.

#### Selegiline

Selegiline is a selective MAO-B at low doses and a non-selective MAOI at higher doses; it also induces dopaminergic activity at low doses. This different action, depending on the dose, implies different use: low doses (up to 10 mg/day) for Parkinson's disease and higher doses as antidepressant treatment (Table 1) (55). The antidepressant activity requires >70% inhibition of MAO-A (20). Conversely, low doses of selegiline have the advantage of partial action on MAO-A, allowing for fewer dietary restrictions. The selegiline transdermal patch is used for a major depressive disorder by releasing from 6 to 12 mg every 24 hours; such a formulation allows higher inhibition of MAO-A and MAO-B in the brain, largely bypassing the inhibition of MAO-A in the gut (55, 223), and thus, no dietary restrictions are recommended when low doses are used. No significant increases in systolic BP as a result of “cheese reaction” were observed when high doses of tyramine (75 mg, a load largely exceeding the amount of a normal meal) were tested in subjects administered a selegiline transdermal patch, supporting its safety and tolerability (224, 225). However, other evidence still underlies using caution and the need for dietary restriction when using 9 or 12 mg/day *via* patch because of limited clinical and experimental experience (Table 3) (226). Higher doses of oral and transdermal selegiline have been linked to a major frequency of orthostatic hypotension (227). No hypertensive crisis was reported with patch administration, but a small portion of patients with preexisting hypertension showed a worse BP control (224). The same study also showed that almost 10% of patients experienced an orthostatic BP decline (with a drop of 10 mmHg in mean BP) as compared with 6.7% in the placebo group (224), suggesting that especially in the elderly, a close BP monitoring is needed. Liver and kidney disease are not a major concern as no dosage adjustments for selegiline are required (226).

#### Moclobemide and Isocarboxazid

Moclobemide is a selective, reversible inhibitor of MAO-A, so the dietary restriction is not required during treatment (228–230). No significant effects on BP profiles have been reported during moclobemide treatment (124, 230–232). On the other hand, isocarboxazid is a non-selective irreversible MAOI and is considered a risk for developing orthostatic hypotension in a dose-dependent manner, although the evidence is not ample due to its limited clinical use (Table 2) (55).

## Conclusions

This narrative review summarized the available studies on the effects of antidepressants on BP. Antidepressants act on several neurotransmitter systems that have direct or indirect effects on BP regulation. Thus, antidepressant drugs might lead individuals to develop hypertension, hypotension, and orthostatic hypotension through a wide array of mechanisms. Although general considerations can be made on different classes of antidepressants, each compound ultimately shows a very specific profile that should be considered before clinical use. Patient characteristics are another obvious source of variability for the effects of antidepressants on BP. In sum, the neutral effect on BP is a feature of SSRI, and this class represents a safe option for patients with preexisting cardiac disease. The occurrence of orthostatic hypotension should always be considered as a real risk in serotonin agonist/antagonist-reuptake inhibitor-trazodone-prescribed patients, but especially in TCA-prescribed patients, with the highest rate in those receiving imipramine and the lowest with nortriptyline. As with orthostatic hypotension, treatment with SNRI, dopamine–NE reuptake inhibitor, and MAOI should be carefully monitored in patients with preexisting hypertension due to their potential to raise BP and facilitate the incidence of hypertensive crisis. When prescribing an antidepressant, the clinician must carefully evaluate its risk/benefit ratio by taking into account the presence of cardiovascular and cerebrovascular diseases, as well as monitoring the effects of the chosen agents. Consideration must be given that an appropriate treatment for depression can ultimately reduce morbidity and mortality. Finally, SSRIs and other recent antidepressants rarely affect BP in a clinically meaningful way. Nonetheless, careful consideration must be given to patients with preexistent BP abnormalities, the elderly, and to individuals with physical comorbidities. Issues requiring particular attention include potential interactions with antihypertensive agents and factors affecting drug metabolism.

## Author Contributions

ACal, IV, MA, and ACab conceived and designed the review. ACal, IF, IV, MB, PC, ST, MA, and ACab collected the data and contributed to the analysis of literature data. ACal, IF, IV, MB, ST, and ACab performed the analysis of all data. ACal, IV, MB, SZ, RV, PC, MA, and ACab discussion of the results. ACal, IV, MB, and ACab wrote the paper. All authors contributed to the article and approved the submitted version.

## Conflict of Interest

The authors declare that the research was conducted in the absence of any commercial or financial relationships that could be construed as a potential conflict of interest.

## Publisher's Note

All claims expressed in this article are solely those of the authors and do not necessarily represent those of their affiliated organizations, or those of the publisher, the editors and the reviewers. Any product that may be evaluated in this article, or claim that may be made by its manufacturer, is not guaranteed or endorsed by the publisher.

## Footnotes

^1^https://www.medicines.org.uk/EMC/medicine/8386/SPC/Reboxetine/#gref.
